# Association Between Chronic Illness, Masculinity, and Well-Being in Older Black Men

**DOI:** 10.1093/geroni/igaa057.994

**Published:** 2020-12-16

**Authors:** Hana Young, Jessalyn Li, Darlingtina Esiaka

**Affiliations:** 1 Union College, Schenectady, New York, United States; 2 Union College, Union College, New York, United States

## Abstract

Research suggests that living with a chronic illness has deleterious impacts on the well-being and quality of life of aging adults. Specifically, it opposes traditional masculine constructs in men and impacts their physical and mental health outcomes. In this study, we examined the lived experiences of Black men, aged 55 years and over, and diagnosed with chronic illness using life history narratives. Participants responded to open-ended questions such as “what aspects of living with illness do you find relatively difficult or easy?” and “what situations make you particularly aware of your illness?” Common themes that emerged from the participants’ responses were the performance of masculinity, fulfilling family duty and obligation, limited sexual encounters, and feelings of exclusion in one’s community. Additionally, participants stated that chronic illness impacts their mental well-being and triggers behavioral responses that exacerbate their ability to cope with the illness. Their responses highlight the conflict between traditional masculine expectations and the presence of chronic illness and illustrate the extent to which ‘manhood’ is a determinant of health even in older men. Our findings can inform the development of tools and interventions designed to improve the experience of well-being among Black men aging with chronic illness.

